# Habitual tea consumption is associated with a low prevalence of gastroesophageal reflux disease in a Taiwanese population study

**DOI:** 10.7150/ijms.139922

**Published:** 2026-09-03

**Authors:** Cheng-Ming Kuo, Jiun-Hung Geng, Wen-Hung Hsu, Neng-Shen Chu, Szu-Chia Chen

**Affiliations:** 1 Department of Internal Medicine, Kaohsiung Medical University Hospital, Kaohsiung Medical University, Kaohsiung, Taiwan.; 2 Division of Gastroenterology, Department of Internal Medicine, Kaohsiung Medical University Hospital, Kaohsiung Medical University, Kaohsiung, Taiwan.; 3 Department of Urology, Kaohsiung Medical University Hospital, Kaohsiung Medical University, Kaohsiung, Taiwan.; 4 Department of Urology, Kaohsiung Municipal Siaogang Hospital, Kaohsiung Medical University Hospital, Kaohsiung Medical University, Kaohsiung, Taiwan.; 5 School of Medicine, College of Medicine, Kaohsiung Medical University, Kaohsiung, Taiwan.; 6 Department of Internal Medicine, Kaohsiung Municipal Siaogang Hospital, Kaohsiung Medical University Hospital, Kaohsiung Medical University, Kaohsiung, Taiwan.; 7 Division of Nephrology, Department of Internal Medicine, Kaohsiung Medical University Hospital, Kaohsiung Medical University, Kaohsiung, Taiwan.

**Keywords:** tea consumption, content, frequency and daily intake, gastroesophageal reflux disease, Taiwan Biobank

## Abstract

**Background:**

Gastroesophageal reflux disease (GERD) is a common gastrointestinal condition associated with significantly impaired quality of life. While tea consumption has been associated with various health benefits, its relationship with GERD remains controversial. The aim of this study was to evaluate the relationship between the prevalence of GERD and tea consumption, including the type, drinking frequency and daily intake of tea, in a large Taiwanese cohort.

**Methods:**

The type of tea was categorized as fully fermented, semi-fermented, and non-fermented; frequency and daily intake were recorded. Standardized self-reported questionnaires were used to record GERD status. Multivariable logistic regression was used to examine the association between tea consumption and GERD.

**Results:**

Of the 27,119 participants, 4706 (17.4%) had GERD and 22,413 did not. A significant association was found between habitual tea consumption and a low prevalence of GERD (odds ratio [OR], 0.843; 95% confidence interval [CI], 0.779 - 0.911; *p* < 0.001) compared to non-drinkers. However, this association was only identified for semi- and non-fermented teas (OR, 0.832; 95% CI, 0.765 - 0.905; *p* < 0.001), but not fully fermented tea. Daily consumption of 1-2 cups (350-700 mL) (OR, 0.881 and 0.765, respectively) was significantly associated with a low prevalence of GERD, however drinking more than 3 cups per day was not significantly associated with GERD prevalence. Furthermore, drinking tea every day (OR, 0.847) was significantly associated with a low prevalence of GERD, while only drinking tea weekly or monthly was not.

**Conclusion:**

The consumption of semi-fermented and non-fermented tea was associated with a low prevalence of GERD, but the association depended on the frequency and quantity of consumption. Further studies are needed to investigate the biological mechanisms of different types of tea and establish interventional strategies for those at high risk of GERD.

## Introduction

Gastroesophageal reflux disease (GERD) is a chronic, relapsing disorder characterized by the retrograde flow of gastric contents into the esophagus, leading to bothersome symptoms and potential mucosal damage 1, 2. GERD is one of the most prevalent clinical gastrointestinal disorders globally, with a pooled worldwide prevalence of approximately 13%, while the prevalence in regions such as North America approaches 20% 3-5. The disease exerts a substantial burden on healthcare systems and significantly impairs a patient's quality of life. The pathophysiology of GERD is multifactorial, primarily driven by failure of the physiological anti-reflux barrier to protect the esophageal mucosa 1, 2. The dominant mechanism for reflux events involves transient lower esophageal sphincter relaxations (tLESRs), which occur independently of the swallowing reflex 2, 6. The clinical presentation of GERD is highly heterogeneous 7-12. Typical symptoms include classical heartburn and acid regurgitation, often exacerbated by specific dietary triggers or the supine position. However, patients frequently present with atypical or extraesophageal manifestations, such as noncardiac chest pain, chronic cough, asthma, and sleep disturbances 13. Without effective treatment, GERD can progress to severe complications including erosive esophagitis, peptic strictures and Barrett's esophagus, and sometimes even hemorrhage and perforation 14.

Tea is widely consumed worldwide, and its beneficial anti-aging 15, metabolic, anti-obesity 16, and antiviral 17 health effects have been extensively studied. Tea consumption has also been associated with potentially reduced risks of various diseases including diabetes 18, cardiovascular disease 19, liver disorders 20, and certain cancers, including prostate 21, oral 22, esophageal 23, nasopharyngeal 24, lung 25, ovarian 26 and bladder cancer 27. However, a previous study suggested that drinking tea may increase the risk of GERD in East Asian populations, while decreasing the risk in Middle Asian populations 28. In addition, a previous prospective study did not find an association between drinking coffee or tea with reflux symptoms or erosive esophagitis; however, drinking coffee with milk was associated with reflux symptoms and drinking "tea and coffee" was associated with erosive esophagitis in univariate analysis. The study concluded that the stimulation of gastric acid secretion or esophageal sensitivity to low pH (or perhaps hyperosmolar solutions) may account for these symptoms 29.

The aim of the present study was to evaluate the association between the prevalence of GERD and tea consumption, including the type, drinking frequency and daily intake of tea using data from 27,119 participants in the Taiwan Biobank (TWB). We hypothesized that tea consumption may be associated with a lower prevalence of GERD. The findings of this study may provide a more comprehensive understanding of the relationship between tea consumption and GERD, and contribute to the development of interventional strategies.

## Materials and Methods

### Identification of the study cohort

Of the 27,209 TWB enrollees, 90 were excluded due to missing tea consumption data, leaving a final study cohort of 27,119 participants (mean age 55.0±10.3 years; 17,530 females; 9589 males) (Figure 1).

The TWB includes the medical, genetic, and lifestyle information of Taiwanese adults between the ages of 30 and 70 years, none of whom have been diagnosed with cancer 30, 31. The aim of the TWB, which is managed by the Ministry of Health and Welfare, is to strengthen health care in Taiwan, considering the challenges of chronic diseases and the aging population. The establishment and operation of the TWB are overseen by both the Ethics and Governance Council of the TWB and Institutional Review Board (IRB) on Biomedical Science Research at Academia Sinica.

Following written informed consent, data were gathered from each participant via in-person interview, physical examination, and blood testing. This included tobacco and alcohol history, diabetes mellitus and hypertension status, sex, age, height and weight. Laboratory measures included fasting glucose, uric acid, hemoglobin, triglycerides and total cholesterol. The estimated glomerular filtration rate (eGFR) was determined with the 2021 Chronic Kidney Disease Epidemiology Collaboration creatinine equation 32.

Blood pressure (BP) was measured by trained personnel, and the mean of three systolic and diastolic readings taken after 1-2-minute rest intervals were recorded. The participants abstained from nicotine, caffeine, and physical exercise for a minimum of 30 minutes prior to all measurements. Physical activity levels were also assessed; regular exercise was defined as a minimum of three 30-minute sessions per week 33. This study adhered to the principles of the Declaration of Helsinki, and the IRB of Kaohsiung Medical University Hospital granted ethical approval (KMUHIRB-E(I)-20210058, 23 March 2023).

### Assessment of tea consumption

The participants were classified into two groups according to whether they were habitual or non-habitual tea drinkers. The habitual tea drinkers were asked the following questions to gather information on the type, daily intake, and frequency of tea consumption:

1. “What type of tea do you usually drink?” Their responses were used to categorize them as drinkers of “fully fermented”, “semi-fermented” or “non-fermented” tea. Fully fermented tea was typically represented by black tea; semi-fermented tea by oolong tea; and non-fermented tea by green tea and white tea.

2. “How many cups of tea do you typically drink each day?” According to their responses, daily intake was grouped as “three or more”, “two”, “one”, or “no” cups per day. One cup was defined as approximately 350 mL.

3. “How frequently do you drink tea?” Their responses were used to categorize them into “monthly” (frequency less than weekly), “weekly” (frequency less than daily), “daily”, and “never” subgroups.

### Definition of GERD

The participants were asked if they had been diagnosed with GERD. Those who responded “Yes” were classified into the GERD group.

### Statistical analysis

Statistical analysis was conducted with SPSS version 25 (IBM Inc., Armonk, NY). Data are presented as number with percentage or mean ± SD. Continuous and categorical variables were compared using the independent t and chi-square test, respectively. Associations among tea consumption, frequency, intake and type of tea with GERD were evaluated in multivariable logistic regression analysis. Confounders were selected a priori on the basis of established or biologically plausible determinants of GERD. Multicollinearity was assessed by calculating variance inflation factors for all covariates, with particular attention to hypertension and systolic BP. Tests for trend were performed by entering daily intake (coded 0, 1, 2 and 3 for none, one, two, and three or more cups per day) and frequency of consumption (coded 0, 1, 2 and 3 for never, monthly, weekly and daily) as single ordinal terms. The analyses of habitual tea consumption, tea type, daily intake and frequency were pre-specified and follow directly from the stated aim of the study. Subgroup analyses stratified by sex, age (< 65 and ≥ 65 years) and hypertension status were exploratory and were not pre-specified; tests for interaction were performed by entering multiplicative interaction terms into the full model. A *p* value < 0.05 was considered to indicate a statistically significant difference.

## Results

### Differences in clinical characteristics between the GERD and non-GERD groups

Of the 27,119 participants, 4706 (17.4%) and 22,413 (82.6%) were classified into the GERD and non-GERD groups, respectively. The GERD group was older, had more female participants, higher rates of hypertension, regular exercise and ever being married, lower rate of tea and coffee consumption, and different education status compared to the non-GERD group. In addition, the GERD group had lower systolic and diastolic BP measurements, higher total cholesterol, and lower eGFR than the non-GERD group (Table 1).

### Association between tea consumption and GERD

All variance inflation factors were below 2.139 (hypertension, 1.271; systolic blood pressure, 1.374), indicating no material multicollinearity. The results of multivariable logistic analysis after adjusting age, sex, diabetes mellitus, hypertension, smoking and alcohol history, coffee consumption, regular exercise habit, married status, education status, systolic BP, body mass index, total cholesterol and eGFR to examine the association between tea consumption and GERD are shown in Table 2. The results showed that older age (*p* < 0.001), female (*p* < 0.001), hypertension (*p* < 0.001), smoking history (*p* < 0.001), junior / senior high school (*vs.* elementary school and below;* p* = 0.021), and high total cholesterol (*p* = 0.034) were significantly associated with a high prevalence of GERD. In contrast, habitual tea consumption (vs. no habitual tea consumption as the reference category) (odds ratio [OR], 0.843; 95% confidence interval [CI], 0.779-0.911; *p* < 0.001), coffee drinkers (*p* < 0.001), and high systolic BP (*p* < 0.001) were significantly associated with a low prevalence of GERD.

### Associations among the type, intake and frequency of tea consumption with GERD

In Table 1, multivariable logistic regression analysis was performed to examine the associations among the type, intake and frequency of tea consumption with GERD (Table 3). We analyzed 3 type of tea (non-tea, fully-fermented tea and semi- or non-fermented tea), the results revealed that drinking non- or semi-fermented tea (*vs.* non-tea drinkers; OR, 0.832; 95% CI, 0.765-0.915; *p* < 0.001) was significantly associated with low GERD prevalence. However, drinking fully fermented tea (*p* = 0.288) was not significantly associated with GERD. Furthermore, we analyzed 4 type of tea (non-tea, fully-fermented tea, semi-fermented tea and non-fermented tea), drinking semi-fermented tea (*vs.* non-tea drinkers; OR, 0.840; 95% CI, 0.759-0.929; *p* = 0.001), and drinking non-fermented tea (*vs.* non-tea drinkers; OR, 0.819; 95% CI, 0.720-0.932; *p* = 0.003) were significantly associated with low GERD prevalence.

The ORs for GERD based on tea consumption frequency are shown in Table 3. Compared to those who were not regular tea drinkers (no cups per day), those with daily consumption of one (OR, 0.881; 95% CI, 0.778-0.998; *p* = 0.046) and two cups (OR, 0.765; 95% CI, 0.676-0.866; *p* < 0.001) were significantly associated with low GERD prevalence. However, those with a daily consumption of three or more cups (*p* = 0.084) were not significantly associated with GERD. The test for linear trend across intake categories, *p* for trend < 0.001.

With regards to tea consumption frequency, the participants with daily consumption (OR, 0.847; 95% CI, 0.781- 0.918; *p* < 0.001) were significantly associated with low GERD prevalence compared to those without regular tea consumption. However, those with a frequency of weekly (*p* = 0.154) and monthly (*p* = 0.198) were not significantly associated with GERD. The test for linear trend across frequency categories, *p* for trend < 0.001.

### Associations among tea consumption and GERD in subgroup analyses

To adjust for baseline differences between the habitual tea drinkers and non-habitual tea drinkers, we conducted subgroup analyses to further evaluate associations among types of tea and GERD (Table 4). Male (OR: 0.862, 95% CI [0.761- 0.976];* p* = 0.019) and female (OR: 0.814, 95% CI [0.726 - 0.910]; *p* < 0.001) semi- or non-fermented tea also had a low prevalence of GERD compared to non-habitual tea drinkers. Among those age < 65 years (OR: 0.848, 95% CI [0.772 - 0.931]; *p* = 0.001) and age ≥ 65 years (OR: 0.760, 95% CI [0.629 - 0.918]; *p* = 0.004), semi- or non-fermented tea also had a low prevalence of GERD compared to non-habitual tea drinkers. In addition, Among the participants without (OR: 0.836, 95% CI [0.761 - 0.920]; *p* < 0.001) and with hypertension (OR: 0.816, 95% CI [0.681 - 0.977]; *p* = 0.027), semi- or non-fermented tea also had a low prevalence of GERD compared to non-habitual tea drinkers. However, the interaction analysis between types of tea and all variables on GERD did not achieve significance.

## Discussion

The aim of this population-based study including a large cohort from the TWB was to elucidate the relationship between habitual tea consumption and the prevalence of GERD. The results showed that those who habitually drank tea had a significantly lower prevalence of GERD, and that the inverse association was significant for semi- and non-fermented teas. Daily tea consumption of one to two cups (estimated at 350-700 mL) showed a significant association with lower GERD prevalence, while higher intake did not. The frequency of tea consumption also played an important role; only daily consumption was associated with a significantly lower GERD prevalence, whereas weekly or monthly consumption was not.

The first notable finding is that drinking semi- and non-fermented teas was associated with a low prevalence of GERD. The relationship between tea consumption and GERD has historically been controversial. A previous meta-analysis of 30 studies including those with a cross-sectional design evaluated the association between tea consumption and GERD across different populations. Although the overall results showed no significant correlation (OR = 1.12), subgroup analyses indicated that drinking tea increased GERD risk in asymptomatic individuals and East Asian populations (OR = 1.27, 95% CI = 1.07-1.51), while decreasing the risk in Middle Asian groups (OR = 0.77, 95% CI = 0.63-0.95) 34. Our findings challenge the broad generalization that tea inherently exacerbates reflux in East Asian individuals. While previous studies have not made detailed distinctions regarding the type of tea (fermented or not) or drinking frequency, our study incorporates these details, potentially offering more nuanced data on these variables. A prospective study conducted at the University of Texas Southwestern Medical School at Dallas utilized *in vitro* laboratory measurements of beverage acidity and osmolality along with a questionnaire assessing postprandial heartburn severity in 394 symptomatic patients. The results suggested that drinking coffee or tea was not associated with true reflux or erosive esophagitis, and that the stimulation of gastric acid secretion or esophageal sensitivity to a low pH may instead account for the reported symptoms (*r* = 0.82; *p* < 0.001) 29. Another study combined *in vitro* bacterial cultures and an *in vivo* mouse model to evaluate the antibacterial efficacy of green tea against *Helicobacter* species. The results demonstrated that green tea exhibited strong dose-dependent bactericidal properties, and that its consumption either before or after infection effectively prevented or significantly reduced gastric inflammation in mice 35, 36. The differences in previous studies likely stem from treating tea as a single dietary factor, failing to account for the substantial biochemical differences between tea preparation methods and the biphasic dose-response of its active compounds. By stratifying tea consumption by fermentation status and volume, our data provide a more granular understanding of this dietary interaction. The differential effects observed between non- and semi-fermented teas (e.g., green and oolong tea) and fully fermented tea (e.g., black tea) can likely be attributed to their distinct phytochemical profiles. Non- and semi-fermented teas are rich in unoxidized polyphenols, predominantly catechins such as epigallocatechin gallate 37. Polyphenols are a subclass of antioxidants that have been shown to have potential therapeutic benefits for GERD 38. As highlighted in a recent pathophysiological study, GERD-induced tissue injury is not solely the result of caustic chemical burns from gastric acid, but is largely driven by cytokine-mediated inflammatory pathways triggered by refluxate exposure 39. The high antioxidant capacity of the polyphenols found in non- and semi-fermented teas may exert a localized anti-inflammatory effect on the esophageal mucosa 28, 40. By scavenging reactive oxygen species and downregulating pro-inflammatory cytokines, these compounds may increase mucosal resistance, thereby mitigating symptomatic and structural damage, and even reduce the risk of cancer 41. Non-fermented tea, such as green tea, has been shown to have a higher polyphenol content than fermented tea 42. Fully fermented teas undergo extensive enzymatic oxidation during processing, which converts these protective catechins into theaflavins and thearubigins, potentially reducing their localized anti-inflammatory efficacy 43. In this study, fully fermented tea did not show a significant association, possibly due to the reduction of active compounds during the fermentation process attenuating its potential health benefits. Further investigations are warranted to elucidate these underlying mechanisms. Because semi-fermented and non-fermented teas both retain a substantial proportion of unoxidized catechins, they were combined in the primary analysis, the intended contrast being between oxidized and largely unoxidized tea polyphenols; we acknowledge, however, that oolong and green or white teas differ in both phytochemical and caffeine profile, and separate estimates are therefore also reported, semi-fermented and non-fermented were also separately associated with a low prevalence of GERD.

Our results also indicated a possible association between the quantity of tea consumed and GERD. The participants with daily consumption of one to two cups showed a significant association with GERD, whereas those with a daily consumption of three or more cups did not. One possible explanation, which we advance as a hypothesis rather than as an account of our findings, involves the pharmacological action of the methylxanthines caffeine and theophylline present in all tea types 29, 44. At high systemic concentrations, caffeine is known to stimulate gastric acid secretion and decrease basal lower esophageal sphincter (LES) pressure 45. More critically, methylxanthines may increase the frequency of tLESRs, the dominant motor mechanism facilitating retrograde flow of the "acid pocket" 46. A significant inhibitory effect on visceral pain has also been documented 47. We therefore hypothesize that at a moderate intake of one to two cups (approximately 350-700 mL), the mucosal anti-inflammatory properties of tea polyphenols may outweigh the mild physiological alterations to the LES, whereas at three or more cups the cumulative burden of caffeine-induced tLESRs and increased intragastric pressure may overwhelm any localized mucosal protection. It must be emphasized that this remains a hypothesis and not an explanation of our data: we measured neither caffeine intake, nor polyphenol concentration, nor LES pressure, nor the frequency of tLESRs or reflux episodes, and the proposed balance is inferred entirely from the published literature. Furthermore, the estimate for one cup per day was of borderline statistical significance (95% CI, 0.778-0.998), the estimate for three or more cups per day had a confidence interval crossing unity (95% CI, 0.791-1.015), and the confidence intervals for the three intake categories overlap substantially. The observed pattern is therefore statistically compatible with a single association of similar magnitude across categories, and does not by itself establish a biphasic biological dose-response. In addition, a previous observational study investigated the association between specific dietary habits and asymptomatic erosive esophagitis (AEE) in 832 asymptomatic Taiwanese men undergoing routine upper gastrointestinal endoscopy. The findings indicated that hiatus hernia (adjusted OR (aOR) 5.0, 95% CI 2.6 - 9.6, *p* < 0.0001), consuming alcohol ≥ 4 days/week (aOR 2.3, 95% CI 1.3 - 4.0, *p* = 0.002), and consuming tea ≥ 4 days/week (aOR 1.6, 95% CI 1.1 - 2.3, *p* = 0.008) were independent risk factors, while the concurrent frequent consumption of both beverages compounded the risk by 3.8 times (95% CI 1.7-8.7) 48. Differences in consumption patterns such as frequency and portion size could also contribute to the observed lack of association. These factors warrant cautious interpretation and highlight the need for more detailed data in future studies.

An alternative explanation that our design cannot exclude, and which deserves emphasis, is reverse causation. Rather than tea consumption being associated with a lower prevalence of GERD, individuals who develop reflux symptoms may reduce or stop drinking tea. Reflux symptoms are commonly attributed to caffeinated beverages, and dietary counselling in Taiwan routinely advises patients with reflux to reduce tea, coffee and other caffeinated drinks; a lower prevalence of tea drinking among participants with GERD may therefore reflect behavioral modification after disease onset rather than any effect of tea itself. Our data cannot distinguish between these possibilities, because the TWB records neither the date of GERD diagnosis nor the duration or history of tea consumption, so the analysis cannot be restricted to exposure that demonstrably preceded the outcome. We note one observation that does not fit neatly with a purely behavioral explanation: if symptomatic avoidance accounted for the entire association, a monotonic gradient would be expected, with the heaviest tea drinkers showing the lowest prevalence of GERD, whereas we observed an association at one to two cups per day that was attenuated and non-significant at three or more cups per day. This is offered as an observation only, and not as evidence against reverse causation, since selective avoidance could plausibly operate in a non-linear fashion. A definitive assessment requires a longitudinal analysis restricted to participants free of GERD at baseline using the TWB follow-up waves, and we regard this as the necessary next step.

The magnitude of the associations we report should also be placed in context. Although the p values are small, the effect sizes are modest. An adjusted OR of 0.843 for habitual tea consumption corresponds, on the absolute scale, to a crude prevalence of GERD of approximately 15.4% among habitual tea drinkers compared with approximately 18.0% among non-drinkers, an absolute difference of about 2.6 percentage points and a number needed to be exposed of roughly 38. An association of this size is modest at the level of the individual patient and is not by itself a basis for dietary advice, although a shift of two to three percentage points in the prevalence of a condition affecting approximately one in six adults is not negligible at the population level. With 27,119 participants, confidence intervals are narrow and *p* values small even for associations of limited clinical consequence, and statistical significance should therefore not be read as a measure of clinical importance.

There are important strengths to this study, including the large community-based sample of healthy individuals with no history of cancer, and controlling for confounding factors. However, there are also several limitations. First, self-reported GERD was assessed using a questionnaire without verification, and the type or severity of GERD could not be obtained. Second, and most importantly, the cross-sectional design precludes any inference about temporal sequence, and reverse causation remains a serious alternative explanation, as discussed above. Third, residual confounding cannot be excluded. Although body mass index, smoking, alcohol consumption and diabetes mellitus were included a priori in the primary model, the TWB does not record the use of proton pump inhibitors, H2-receptor antagonists or antacids, the use of NSAIDs or aspirin, Helicobacter pylori status, the presence of hiatal hernia, meal timing and late-night eating, or detailed dietary patterns such as the intake of fatty or spicy food. Several of these are strong determinants of GERD, and acid-suppressive medication in particular lies on the causal pathway to symptom control rather than being a simple confounder, which complicates the interpretation of a self-reported diagnosis in either direction. Our estimates should therefore be regarded as adjusted associations rather than as unbiased causal effects. Fourth, the assessment of tea exposure was crude. The TWB questionnaire records the predominant type of tea, the number of cups per day and the frequency of consumption, but not the leaf-to-water ratio or brewing strength, steeping time, serving temperature, measured caffeine or polyphenol content, additives such as sugar or milk, the number of years of tea drinking, or changes in tea type over time. Serving temperature deserves particular emphasis, since very hot beverages are themselves associated with esophageal mucosal injury. In addition, one cup was defined as approximately 350 mL for the purposes of the questionnaire and was not measured, so daily intake is best regarded as an ordinal ranking rather than a quantitative dose; for the same reason we could not determine the intake above which the inverse association with prevalent GERD is no longer observed. Fifth, although the analyses of tea type, daily intake and frequency were pre-specified, no adjustment was made for multiple comparisons. A Bonferroni correction across the comparisons presented in Table 3 would set the significance threshold at approximately α = 0.006; on this criterion the associations for semi-/non-fermented tea, for two cups per day and for daily consumption would remain significant, whereas the borderline association for one cup per day (p = 0.046) would not. The latter should therefore be regarded as hypothesis-generating, and type I error cannot be excluded for the borderline comparisons. Sixth, it is common to distinguish different types of tea according to how the tea is fermented, and to use the classification of fully, semi- and non-fermented tea. However, there are still subtle differences between the fermentation types, including the tea tree and place of origin. Finally, the participants in this study were of Chinese ethnicity, and thus our findings may not be generalizable to other ethnicities.

## Conclusions

This study identifies an inverse association between tea consumption and the prevalence of GERD, which was most evident for daily, moderate intake of semi-fermented and non-fermented teas. Given the cross-sectional nature of this study, however, no causal link between tea consumption and GERD can be established, and reverse causation, a reduction in tea intake following the onset of reflux symptoms, remains a plausible alternative explanation. Prospective research restricted to individuals free of GERD at baseline is needed to assess the risk of developing new-onset GERD over time. In addition, since the definition of GERD in the TWB relies on self-reported, further studies using validated symptom instruments or endoscopically confirmed diagnoses are warranted to identify other contributing factors and to better characterize the relationship between tea consumption and GERD in different populations.

## Figures and Tables

**Figure 1:**
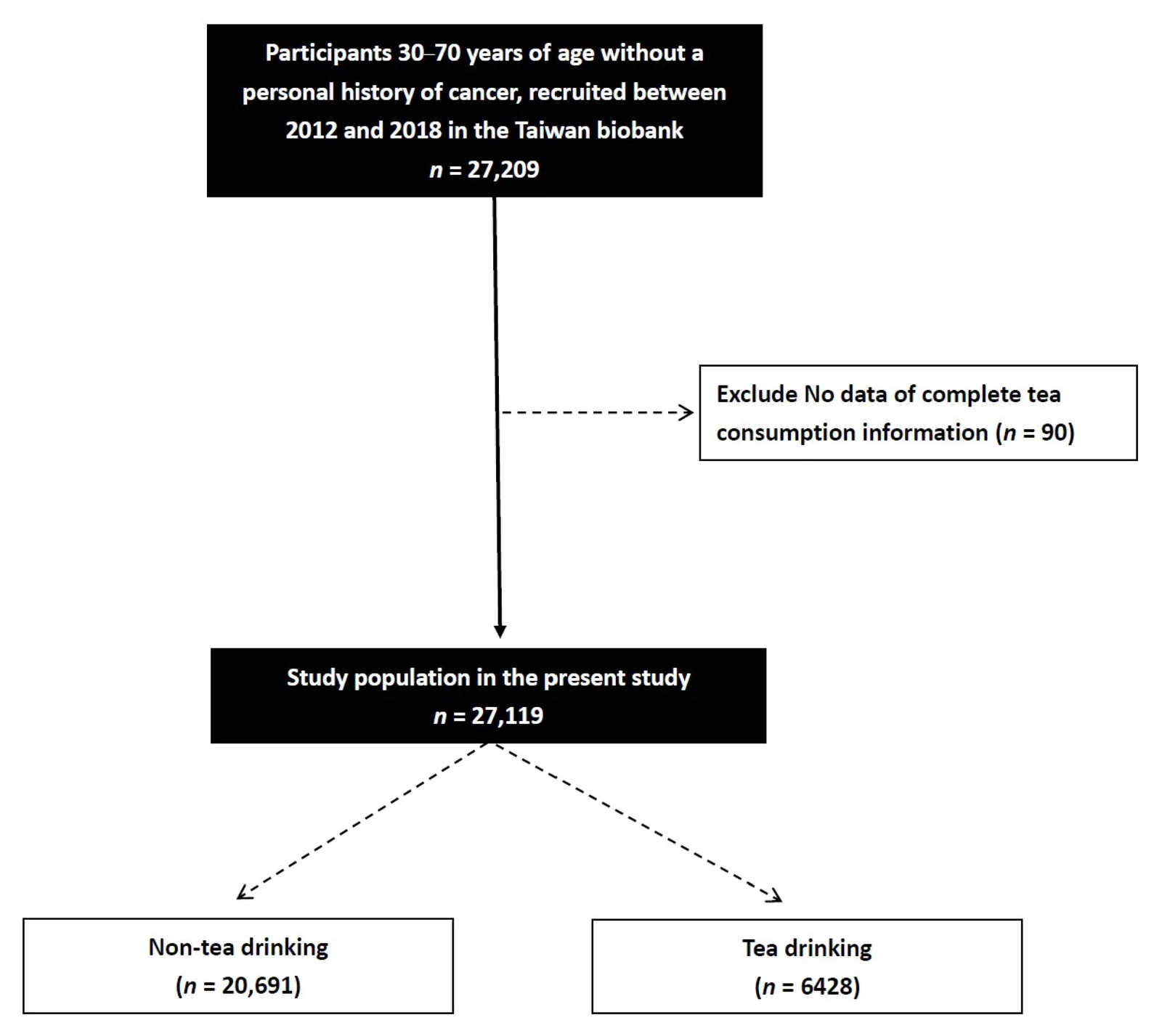
Flowchart of study population.

**Table 1:** Comparison of clinical characteristics among participants according to GERD in study participants

Characteristics	GERD (-) (*n* = 22,413)	GERD (+) (*n* = 4706)	*p*
Age (year)	54.8 ± 10.4	56.3 ± 9.6	< 0.001
Male sex (%)	35.8	33.0	< 0.001
Diabetes mellitus (%)	7.6	8.4	0.052
Hypertension (%)	17.3	20.7	< 0.001
Smoking history (%)	25.5	26.0	0.528
Alcohol history (%)	10.6	10.9	0.561
Habitual tea consumption (%)	24.3	21.0	< 0.001
Habitual coffee consumption (%)	41.6	38.9	< 0.001
Regular exercise habits (%)	47.8	50.7	< 0.001
Ever married (%)	92.3	93.8	< 0.001
Education status			< 0.001
Elementary school and below (%)	7.3	7.6	
Junior / senior high school (%)	43.3	46.6	
University or above (%)	49.4	45.8	
Systolic BP (mmHg)	125.0 ± 19.7	123.9 ± 18.3	< 0.001
Diastolic BP (mmHg)	74.3 ± 11.5	73.8 ± 10.9	0.001
Body mass index (kg/m^2^)	24.3 ± 3.7	24.3 ± 3.7	0.131
Laboratory parameters			
Fasting glucose (mg/dL)	97.4 ± 21.9	97.4 ± 20.7	0.947
Hemoglobin (g/dL)	13.7 ± 1.6	13.7 ± 1.5	0.425
Triglyceride (mg/dL)	120.0 ± 99.7	120.6 ± 82.6	0.652
Total cholesterol (mg/dL)	196.0 ± 36.2	197.1 ± 36.2	0.049
eGFR (mL/min/1.73 m^2^)	98.2 ± 15.1	97.0 ± 15.2	< 0.001
Uric acid (mg/dL)	5.4 ± 1.4	5.4 ± 1.3	0.051

Data are presented as percentage or mean ± SD. Comparisons between groups are unadjusted and were made using the independent t test for continuous variables and the chi-square test for categorical variables. Abbreviations. GERD, gastroesophageal reflux disease; BP, blood pressure; eGFR, estimated glomerular filtration rate.

**Table 2:** Association of tea consumption with GERD using multivariable regression analysis

Variables	Multivariable (GERD)	
	OR (95% CI)	*p*
Age (per 1 year)	1.016 (1.011-1.020)	< 0.001
Male sex (*vs.* female)	0.840 (0.767-0.920)	< 0.001
Diabetes mellitus	1.025 (0.909-1.157)	0.685
Hypertension	1.280 (1.171-1.401)	< 0.001
Smoking history	1.180 (1.076-1.294)	< 0.001
Alcohol history	1.084 (0.970-1.213)	0.155
Habitual tea consumption	0.843 (0.779-0.911)	< 0.001
Habitual coffee consumption	0.906 (0.848-0.967)	0.003
Regular exercise habits	1.026 (0.960-1.098)	0.448
Ever married	1.109 (0.971-1.266)	0.126
Education status		
Elementary school and below	Reference	
Junior / senior high school	1.162 (1.023-1.319)	0.021
University or above	1.121 (0.981-1.280)	0.093
Systolic BP (per 1 mmHg)	0.992 (0.991-0.994)	< 0.001
Body mass index (per 1 kg/m^2^)	1.001 (0.992-1.011)	0.807
Total cholesterol (per 1 mg/dL)	1.001 (1.000-1.002)	0.034
eGFR (per 1 mL/min/1.73 m^2^)	0.999 (0.997-1.002)	0.711

Values expressed as odds ratio (OR) and 95% confidence interval (CI). Abbreviations are the same as in Table 1. Adjusted for age, sex, diabetes mellitus, hypertension, smoking and alcohol history, coffee consumption, regular exercise habit, married status, education status, systolic BP, body mass index, total cholesterol and eGFR.

**Table 3:** Association of content, daily cups and frequency of tea consumption with GERD using multivariable regression analysis

Tea consumption	GERD status, n (%)		Multivariable (GERD)	
	GERD (-)	GERD (+)	OR (95% CI)	*p*
Type of tea (3 types)	‍	‍		
Non-tea	16973 (75.7%)	3718 (79.0%)	Reference	
Fully-fermented tea	800 (3.6%)	150 (3.2%)	0.910 (0.761-1.089)	0.302
Semi- or non-fermented tea	4640 (21.0%)	838 (17.8%)	0.832 (0.765-0.905)	< 0.001
Type of tea (4 types)				
Non-tea	16973 (75.7%)	3718 (79.0%)	Reference	
Fully-fermented tea	800 (3.6%)	150 (3.2%)	0.910 (0.761-1.088)	0.302
Semi-fermented tea	2919 (13.0%)	540 (11.5%)	0.840 (0.759-0.929)	0.001
Non-fermented tea	1721 (7.7%)	298 (6.3%)	0.819 (0.720-0.932)	0.003
Daily cups				
None	16973 (75.7%)	3718 (79.0%)	Reference	
1 cup* per day	1699 (7.6%)	331 (7.0%)	0.881 (0.778-0.998)	0.046
2 cups* per day	1987 (8.9%)	325 (6.9%)	0.765 (0.676-0.866)	< 0.001
≥ 3 cups* per day	1754 (7.8%)	332 (7.1%)	0.896 (0.791-1.015)	0.084
*p* for trend				< 0.001
Frequency				
None	16973 (75.7%)	3718 (79.0%)	Reference	
Per day	5015 (22.4%)	916 (19.5%)	0.847 (0.781-0.918)	< 0.001
Per week	406 (1.8%)	71 (1.5%)	0.830 (0.642-1.072)	0.154
Per month	19 (0.08%)	1 (0.2%)	0.266 (0.036-1.993)	0.198
*p* for trend	‍	‍	‍	< 0.001

Values expressed as odds ratio (OR) and 95% confidence interval (CI). Abbreviations are the same as in Table 1. Adjusted for age, sex, diabetes mellitus, hypertension, smoking and alcohol history, coffee consumption, regular exercise habit, married status, education status, systolic BP, body mass index, total cholesterol and eGFR. * One cup = 0.350 liter

**Table 4:** Subgroup analyses of the association between type of tea and GERD, stratified by sex, age group and hypertension status

Subgroup	No. of participants	No. with GERD, n (%)	Adjusted OR (95% CI) vs. non-tea		*p* for interaction
			Fully fermented tea	Semi- or non-fermented tea	
Sex					0.447
Male	9589	1554 (16.2%)	0.738 (0.537-1.015), *p* = 0.062	0.862 (0.7610.976), *p* = 0.019	
Female	15730	3152 (20.0%)	1.027 (0.826-1.277), *p* =0.809	0.814 (0.726-0.910), *p* < 0.001	
Age group					0.190
< 65 years	21563	3685 (17.1%)	0.908 (0.748-1.104), *p* =0.334	0.849 (0.537-1.341), *p* = 0.482	
≥ 65 years	5556	1021 (18.4%)	0.848 (0.772-0.931), *p* = 0.001	0.760 (0.629-0.918), *p* = 0.004	
Hypertension					. 0.630
Absent	22271	3730 (16.7%)	0.850 (0.695-1.040), *p* = 0.114	0.836 (0.761-0.920), *p* < 0.001	
Present	4848	976 (20.1%)	1.201 (0.806-1.790), *p* = 0.367	0.816 (0.681-0.977), *p* = 0.027	

Values expressed as odds ratio (OR) and 95% confidence interval (CI). Abbreviations are the same as in Table 1. Adjusted for age, sex, diabetes mellitus, hypertension, smoking and alcohol history, coffee consumption, regular exercise habit, married status, education status, systolic BP, body mass index, total cholesterol and eGFR.

## Data Availability

The data underlying this study are from the Taiwan Biobank. Due to restrictions placed on the data by the Personal Information Protection Act of Taiwan, the minimal data set cannot be made publicly available. Data may be available upon request to interested researchers. Please send data requests to: Szu-Chia Chen, PhD, MD. Division of Nephrology, Department of Internal Medicine, Kaohsiung Medical University Hospital, Kaohsiung Medical University.
